# Supplementary material for: Structure and function of the metagenomic plastic-degrading polyester hydrolase PHL7 bound to its product
Source: Nat Commun. 2023 Apr 5;14:1905. doi: 10.1038/s41467-023-37415-x (PMC10076380; doi:10.1038/s41467-023-37415-x)
Supplement: Supplementary file 1 — Supporting Information [file 41467_2023_37415_MOESM1_ESM.pdf]

Supplementary Information

Structure and function of the metagenomic plastic-degrading polyester hydrolase PHL7 bound to its product

P. Konstantin Richter[1], Paula Blázquez-Sánchez[2], Ziyue Zhao[2], Felipe Engelberger[3], Christian Wiebeler[2,4] Georg Künze[3], Ronny Frank[5], Dana Krinke[5], Emanuele Frezzotti[6], Yuliia Lihanova[2], Patricia Falkenstein[2], Jörg Matysik[2], Wolfgang Zimmermann[2*], Norbert Sträter[1*], Christian Sonnendecker[2*]

[1] Institute of Bioanalytical Chemistry, Centre for Biotechnology and Biomedicine, Leipzig University, 04103 Leipzig (Germany)

[2] Institute of Analytical Chemistry, Leipzig University, 04103 Leipzig (Germany)

[3] Institute for Drug Discovery, Leipzig University Medical School, 04107 Leipzig (Germany)

[4] Wilhelm-Ostwald-Institute for Physical and Theoretical Chemistry, Leipzig University, 04103 Leipzig (Germany)

[5] Centre for Biotechnology and Biomedicine, Molecular Biological-Biochemical Processing Technology, Leipzig University, 04103 Leipzig (Germany)

[6] Department of Chemical Life and Environmental Sciences, University of Parma, 43121 Parma (Italy)

*Corresponding authors: wolfgang.zimmermann@uni-leipzig.de, strater@bbz.uni-leipzig.de, christian.sonnendecker@uni-leipzig.de

Supplementary Methods:

Classical Molecular Dynamics

Molecular dynamics (MD) simulations of PHL7 (PDB 7NEI) were carried out using AMBER20 along with the ff19SB force field[1] for protein atoms and the GAFF force field[2] for the EMT ligand atoms. The protonation state of the residues at pH 8.0 was estimated using the H++ server[3]. Then, the MD system was solvated with TIP3P water molecules and neutralized with counterions in a truncated octahedral box of 1.3 nm of padding with periodic boundary conditions. The system was first minimized using the steepest descent method with position restraints applied on waters and ions, followed by a second minimization without any position restraints. The system was heated from 0 to 298 K for 75 ps at a constant volume using a Langevin thermostat at 1 fs time step, followed by equilibration of the solvent and ligand atoms, of each system for 2.5 ns at 298 K with a restraint weight of 10 kcal/mol/Å^2 on all protein atoms at the constant pressure of 1 bar using a Berendsen barostat and a 2 fs time step. Subsequently, a similar equilibration step was performed with restraints applied only on the protein backbone atoms (2.5 ns at 2fs time step and a restraint weight of 10 kcal/mol/Å^2) before the whole system was equilibrated for 2.5 ns under the same temperature and pressure conditions without restraints. Production MD runs were carried out in four replicas for 100 ns each, using a timestep of 2 fs and the SHAKE[4] algorithm to constrain all bonds involving hydrogens. The particle mesh Ewald method[5] was used for long-range electrostatics, with a 10 Å cutoff for short-range electrostatics. The geometry of EMT was optimized with Gaussian 09 (Gaussian, Inc, Wallingford CT) on the B3LYP/6–31G** level of theory, and assignment of AMBER atom types and calculation of RESP charges were done with Antechamber[6]. Independent runs were ensured by using random seeds for initial velocities during the equilibration step. Replicas were checked for structural convergence using the overall backbone root-mean-square deviation (RMSD) relative to the first frame. RMSD and per-residue root mean square fluctuations (RMSF) were calculated using CPPTRAJ of AmberTools22[7]. Distance analysis was conducted using MDanalysis[8] and Matplotlib (Matplotlib: A 2D Graphics Environment | IEEE Journals & Magazine) to generate figures.

Autodock VINA

Control docking calculations were carried out following Engelberger et al.[9] using AutoDock Vina[10,11]. For all variants docking was performed inside a volume of 11 × 27 × 11 grid points (1Å spacing between grid points) that comprises both subsites I and II. We used the same starting structures that were employed for Rosetta docking. We added the polar hydrogens for the protein and parameterized the system with Gasteiger charges and atom types using prepare receptor4.py and prepare ligand4.py scripts from MGLtools 1.5.7.

DiffDock

The DiffDock[12] machine learning model was used to predict the binding mode of a pentamer of PET. To generate the prediction we used as input the following smiles string:

OC(=O)c1ccc(cc1)C(=O)OCCOC(=O)c1ccc(cc1)C(=O)OCCOC(=O)c1ccc(cc1)C(=O)OCCOC(=O)c1ccc(cc1)C(=O)OCCOC(=O)c1ccc(cc1)C(=O)OCC

As input structure we used the crystal structure of unliganded PHL7 (PDB 7NEI). To prepare the structure for the machine learning model we used the ESM-2 protein language model to generate the protein embeddings[13] (i.e., the protein structure latent representation). To run inference, we used the provided scripts from the DiffDock, repository (https://github.com/gcorso/diffdock). As arguments we specified 50 inference steps, and 25 samples. From the generated 25 binding poses the rank 4 prediction was chosen for making illustrations because the PET chain in this model aligned with the positions of EMT and TPA molecules in the Rosetta docking model and the PHL7 crystal structure, respectively.

Supplementary Results:

PHL7 crystallization and structure solution

We previously reported the crystal structure of PHL7 WT at 1.3 Å resolution in space group $P2_12_12_1$ (PDB ID: 7NEI)[14]. In this study, we determined two crystal structures of a catalytically inactive variant in which the active site serine 131 was exchanged by alanine (PHL7 S131A).

In an attempt to elucidate binding of PET hydrolysis products to the active site of PHL7 we cocrystallized PHL7 S131A along with the PET building block TPA. Experiments to grow cocrystals of PHL7 with the mono- and bis-2-hydroxyethylene terephthalates MHET and BHET failed: the resulting electron density maps contain either no ligand density at all or density of only the TPA part, lacking the additional ethylene glycol moieties. The structure of PHL7 S131A that was crystallized in a buffer containing 10 mM $MgCl_2$ reveals presence of Mg^{2+} interacting with D246 and five water molecules (see supplementary fig. 1). The Mg^{2+} cocrystal structure also contains weak electron density of a ligand in the active site of chain B as it was cocrystallized with BHET. This additional density was subsequently modeled as TPA. Crystals of both protein structures belong to space group $P2_1$ with two molecules per asymmetric unit.

PET film degradation experiments of PHL7 variants

We constructed 17 single mutants of PHL7 in order to investigate their influence on PET-hydrolytic activity and thermal stability, including the deactivating S131A variant. The enzymatically catalyzed weight loss of PET films was monitored after incubation at 4, 8 and 16 h and 70 °C, if not stated otherwise. Additionally, the degradation rate of selected variants was also analyzed by electrochemical impedance spectroscopy (EIS) as we consider this method to be more reliable when comparing variants with minor changes in their activity[15]. The melting point (T_m) of PHL7 was determined via nano differential scanning fluorimetry (nanoDSF). Variants L210T and L210V completely hydrolyzed the PET film within 16 h, and L210A, L210I, L93F and D233K caused over 97% weight loss. We therefore calculated normalized weight loss activities relative to PHL7 WT at time points of 4 and 8 h (see Fig. 3A).

Supplementary Tables:

Supplementary Table 1: Overview of point mutations introduced into PHL7.

position	functional location	target AA	reference protein	reference	G-PET weight loss (%)[1]			ΔT_m ± SD (rel. to WT PHL7)
					4 h	8 h	16 h	
- (WT)	-	-	-	-	18.5 ± 1.00	40.8 ± 6.10	90.1 ± 2.78	0.00 ± 0.05
S131	catalytic nucleophile	A	-	-	n.d.[2]	n.d.	n.d.	-0.63 ± 0.04
F63	subsite I	Y	*Is*PETase, LCC	16,17	18.4 ± 0.75	29.0 ± 0.71	83.0 ± 0.92	0.56 ± 0.04
		A	*Is*PETase, LCC	16,17	3.0 ± 0.16	6.7 ± 0.16	10.8 ± 0.12	0.07 ± 0.05
M132	subsite I	W	PHL4	14	n.d.	n.d.	2.9 ± 0.38 (24 h)	1.98 ± 0.17
L93	subsite I	F	LCC	17	22.7 ± 0.89	50.2 ± 1.64	97.6 ± 2.00	1.19 ± 0.04
		A	-	-	19.7 ± 0.58	45.4 ± 0.78	92.0 ± 2.17	-0.44 ± 0.04
Q95	subsite I	Y	LCC	17	21.4 ± 0.68	48.4 ± 0.37	94.6 ± 3.38	0.80 ± 0.05
		G	LCC|ICCG	17	16.4 ± 1.34	41.3 ± 1.03	67.7 ± 2.38	1.99 ± 0.04
H185	subsite I	S	*Is*PETase	18	4.0 ± 0.16	7.3 ± 0.36	10.0 ± 0.31	-11.52 ± 0.05
F189	subsite I	I	*Is*PETase	18	10.8 ± 0.49	14.7 ± 0.34	14.7 ± 1.35	-7.10 ± 0.07
H130	subsite IIa	W	*Is*PETase, PHL4-6	14,18	4.8 ± 0.28	8.1 ± 0.05	12.1 ± 0.18	-4.40 ± 0.07
L210	subsite IIa	F	LCC, *Tf*Cut2	17,19	12.9 ± 0.47	28.0 ± 0.65	55.9 ± 2.16	3.05 ± 0.03
		I	LCC|ICCG	17	25.0 ± 0.13	52.9 ± 1.48	99.8 ± 0.33	-0.51 ± 0.06
		A	*Is*PETase,S238A	16,20	23.5 ± 0.94	52.5 ± 1,31	98.0 ± 2.00	0.37 ± 0.02
		S	*Is*PETase	16	20.8 ± 1.16	46.1 ± 1.57	92.7 ± 3.34	0.91 ± 0.05
		V	-	-	25.6 ± 0.58	54.3 ± 1.33	100	-0.21 ± 0.11
		T	-	-	26.3 ± 0.74	57.2 ± 1.54	100	1.36 ± 0.05
D233	metal binding site	K	-	-	25.4 ± 4.25	44.7 ± 6.02	97.6 ± 4.17	0.91 ± 0.10

[1]: All weight loss experiments were conducted at 70 °C, mean values ± SD for n=3 experiments are shown.
[2]: No weight loss was detectable after 24 h.

Supplementary Table 2: Data collection and refinement statistics of the PHL7×TPA and PHL7×Mg^{2+} crystal structures.

	**PHL7×TPA**	**PHL7×Mg^{2+}**
**Data collection**		
Resolution range [Å]	21.57 - 1.7 (1.761 - 1.7)[1]	21.46 - 1.7 (1.761 - 1.7)[1]
Space group	$P2_1$	$P2_1$
Wavelength [Å]	1.5406	1.5406
Temperature [K]	100	100
Unit cell dimensions a, b, c [Å]	52.91, 56.51, 101.74	52.35, 56.07, 100.44
β [°]	94.11	94.01
Reflections	430,814 (32,577)	312,141 (21,198)
Unique reflections	66,097 (6,614)	63,959 (6,341)
Multiplicity	6.5 (4.9)	4.9 (3.3)
Completeness (%)	99.84 (99.97)	99.77 (99.83)
$I/\sigma(I)$	18.33 (2.80)	19.81 (3.02)
R_{merge}	0.07515 (0.4792)	0.05707 (0.3389)
R_{pim}	0.0303 (0.2392)	0.02696 (0.2168)
$CC_{1/2}$	0.998 (0.832)	0.997 (0.838)
Wilson B-factor	13.42	11.93
**Refinement**		
Reflections used in refinement	66,048 (6,612)	63,957 (6,341)
Reflections used for R_{free}	2,978 (323)	2,885 (319)
R_{work}	0.1600 (0.1972)	0.1296 (0.1723)
R_{free}	0.2059 (0.2495)	0.1604 (0.2103)
No. of non-hydrogen atoms:	4,978	4,852
Protein	4,036	4,006
Solvent	910	832
Ligands	56	20
RMS deviations		
Bonds [Å]	0.004	0.008
Angles [°]	0.77	0.96
Ramachandran		
Favored [%]	98.05	98.25
Allowed [%]	1.95	1.75
Outliers [%]	0.00	0.00
Molprobity clashscore	2.74	1.39
Average B-factor	18.27	16.60
Protein	15.49	13.51
Solvent	30.35	31.32
Ligands	25.35	27.62
No. of TLS groups	2	2
PDB ID	8BRB	8BRA

[1]: Values for highest resolution shell in parentheses.

Supplementary Table 3. Rank and score of Autodock Vina predictions and RMSD to the Rosetta predicted structure for each variant.

Variant	AutodockVina Prediction Rank	Autodock Score	RMSD to Rosetta [Å]
L210A	8	-5.1	2.279
L210F	2	-6.7	2.667
L210I	5	-5.1	2.780
L210S	5	-6.3	2.343
L210T	4	-5	2.556
L210V	5	-4.9	2.944
WT	4	-5	2.773

Supplementary Table 4. Score of Autodock Vina for the nine docking structures predicted for each variant. The structure that most closely resembles the Rosetta prediction is marked in yellow.

Variant	Autodock Prediction #	Autodock Score
L210A	1	-5.7
L210A	2	-5.5
L210A	3	-5.4
L210A	4	-5.3
L210A	5	-5.1
L210A	6	-5.1
L210A	7	-5.1
L210A	8	-5.1
L210A	9	-5
L210F	1	-6.8
L210F	2	-6.7
L210F	3	-6.7
L210F	4	-6.6
L210F	5	-6.5
L210F	6	-6.4
L210F	7	-6.4
L210F	8	-6.3
L210F	9	-6.3
L210I	1	-5.4
L210I	2	-5.3
L210I	3	-5.2
L210I	4	-5.2
L210I	5	-5.1
L210I	6	-5.1
L210I	7	-5.1

L210I	8	-5
L210I	9	-5
L210S	1	-6.4
L210S	2	-6.4
L210S	3	-6.4
L210S	4	-6.3
L210S	5	-6.3
L210S	6	-6.2
L210S	7	-6.2
L210S	8	-6.2
L210S	9	-6.2
L210T	1	-5.5
L210T	2	-5.1
L210T	3	-5
L210T	4	-5
L210T	5	-5
L210T	6	-4.9
L210T	7	-4.8
L210T	8	-4.7
L210T	9	-4.7
L210V	1	-5.3
L210V	2	-4.9
L210V	3	-4.9
L210V	4	-4.9
L210V	5	-4.9
L210V	6	-4.9
L210V	7	-4.8
L210V	8	-4.8
L210V	9	-4.6
WT	1	-5.2
WT	2	-5.1
WT	3	-5
WT	4	-5
WT	5	-5
WT	6	-5
WT	7	-5
WT	8	-4.8
WT	9	-4.8

Supplementary Figures:

```
                                             63
PHL7     ----------------------------------MANPYERGPDPTESSIEAVRGPFAVA  26
PHL4     ----------------------------------MENPYERGPDPTTQSVEAARGPYAVS  26
LCC      MDGVLWRVRTAALMAALLALAAWALVWASPSVEAQSNPYQRGPNPTRSALTADG-PFSVA  59
TfCut2   -----------------------------------ANPYERGPNPTDALLEARSGPFSVS  25
IsPETase -------MNFPRASRLMQAAVLGGLMAVSAAATAQTNPYARGPNPTAASLEASAGPFTVR  53
                                              *** ***:**   : *   *::*

                                             63
PHL7     QTTVSRLQADGFGGGTIYYPTDTSQGTFGAVAISPGFTAGQESIAWLGPRIASQGFVVIT  86
PHL4     QITVG-GQFGSYSGGTIYYPTSTADGKFGAVAIAPGFLSFQSSVAWLGPRIASQGFVVMT  85
LCC      TYTVSRLSVSGFGGGVIYYPTG-TSLTFGGIAMSPGYTADASSLAWLGRRLASHGFVVLV  118
TfCut2   EENVSRLSASGFGGGTIYYPR--ENNTYGAVAISPGYTGTEASIAWLGERIASHGFVVIT  83
IsPETase SFTVS--RPSGYGAGTVYYPTN-AGGTVGAIAIVPGYTARQSSIKWWGPRLASHGFVVIT  110
           .*.    ..:..*.:***      . *.:*: **: .   *: * * *:**:****:.

              93 95                                      130 132
PHL7     IDTITRLDQPDSRGRQLQAALDHLRTN-----SVVRNRIDPNRMAVMGHSMGGGGALSAA  141
PHL4     IDTGTIFDQPAQRGDQLLDALDFLTQR-----SSVKDRIDPNRLAVAGWSWGGGGSLEAA  140
LCC      INTNSRFDYPDSRASQLSAALNYLRTSS---PSAVRARLDANRLAVAGHSMGGGGTLRIA  175
TfCut2   IDTITTLDQPDSRAEQLNAALNHMINRA---SSTVRSRIDSSRLAVMGHSMGGGGSLRLA  140
IsPETase IDTNSTLDQPSSRSSQQMAALRQVASLNGTSSSPIYGKVDTARMGVMGWSMGGGGSLISA  170
         *:* : :* * .*. *   **  :        * : ::*. *:.* * * ****:*  *

                                                   185 189
PHL7     ANNTSLEAAIPLQGWHTRKNWSSVRTPTLVVGAQLDTIAPVSSHSEAFYNSLPSDLDKAY  201
PHL4     ADRPSLKAAIPMAGWNLNTNWSRLTTPVLVVGVQNDLIAPVAMHSEPFYNSIRS--EKAY  198
LCC      EQNPSLKAAVPLTPWHTDKTFN-TSVPVLIVGAEADTVAPVSQHAIPFYQNLPSTTPKVY  234
TfCut2   SQRPDLKAAIPLTPWHLNKNWSSVTVPTLIIGADLDTIAPVATHAKPFYNSLPSSISKAY  200
IsPETase ANNPSLKAAAPQAPWDSSTNFSSVTVPTLIFACENDSIAPVNSSALPIYDSMSRN-AKQF  229
          :...*:** *   *.  ..:.   .*.*:.. : * :***   : .:*:.:     * :

                210                       233
PHL7     MELRGASHLVSNTPD---TTTAKYSIAWLKRFVDDDLRYEQFLCPAP-DDFAISEYRSTCPF--   259
PHL4     LELAGGSHFTVTSAN---TPQAKLMISWLKRFVDNDTRYEQFICPGPSRGFSVSEYRSTCPY--   257
LCC      VELDNASHFAPNSNN---AAISVYTISWMKLWVDNDTRYRQFLCNVNDPALSDFRTNNRHCQ--   293
TfCut2   LELDGATHFAPNIPN---KIIGKYSVAWLKRFVDNDTRYTQFLCPGPRDGLFGEVEEYRSTCPF   261
IsPETase LEINGGSHSCANSGNSNQALIGKKGVAWMKRFMDNDTRYSTFACENPNSTRVSDFRTANCS---   290
         :*: ..:*   .  :      .   ::*:* ::*:* **  * *
```

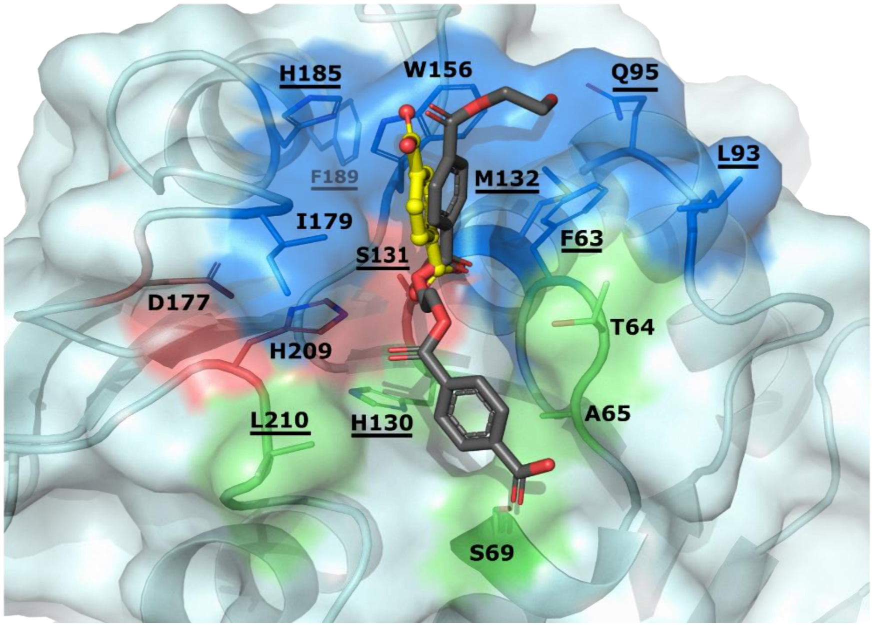

Supplementary Figure 1: Overview of the sites mutated in this study and origin of the chosen replacement residues. A multiple sequence alignment shows the corresponding positions (boxed) selected for mutagenesis and the amino acids to which those positions were mutated (filled residues). The catalytic triad is shown in red, subsite I in blue, subsite II ins green (PHL7 residue numbering). A structural overview of the binding site with the docking model for EMT generated in this study (dark grey) is shown below. Mutation sites are underlined. Superposition of the TPA ligand (yellow) derived from the PHL7×TPA crystal structure (PDB:8BRB) confirms a productive conformation of the docked EMT molecule.

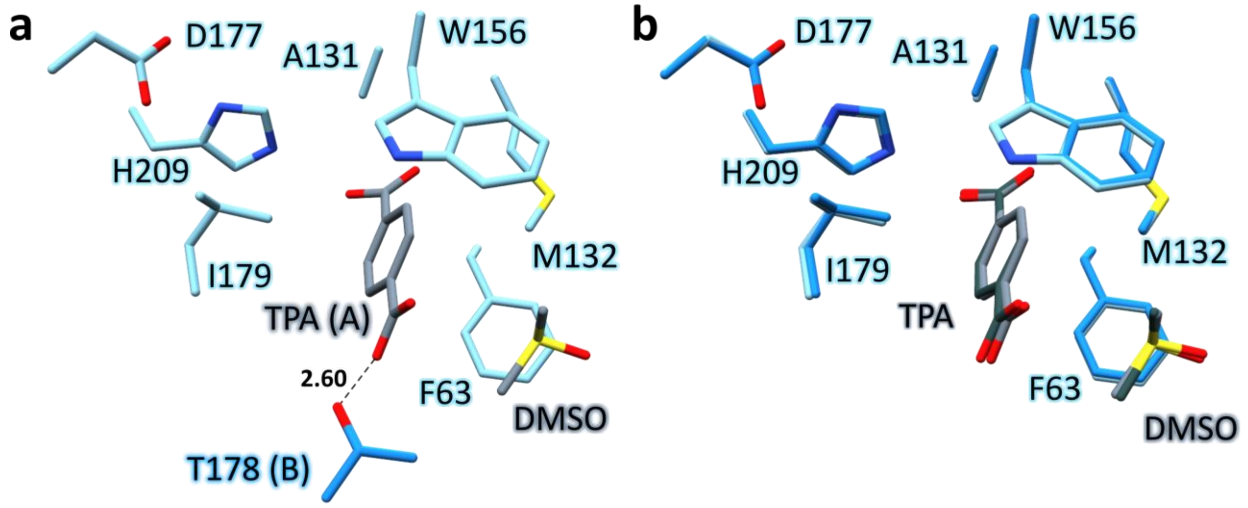

Supplementary Figure 1: Binding of TPA and DMSO to subsite I of PHL7. **a** T178 of chain B (dark blue) interacts with the distal carboxyl group of TPA (grey, distance in Å). **b** Superimposition of the two individual PHL7 chains A (light blue) and B (dark blue) of the PHL7×TPA cocrystal structure. Both the residues as well as the ligands TPA and DMSO (chain A: light grey, chain B: dark grey) align well. Therefore, interaction of T178 of chain B with TPA of chain A does not influence the conformation of the active site.

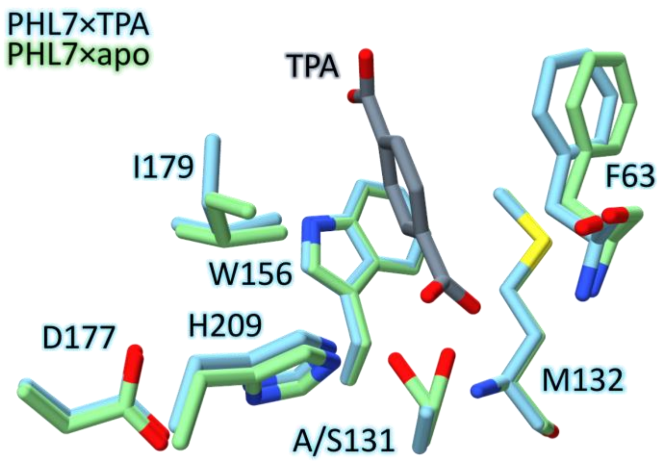

Supplementary Figure 2: Superimposition of subsite I of PHL7×TPA (light blue) and apo PHL7 (light green, PDB ID: 7NEI). Binding of TPA does not significantly alter the conformation of binding residues.

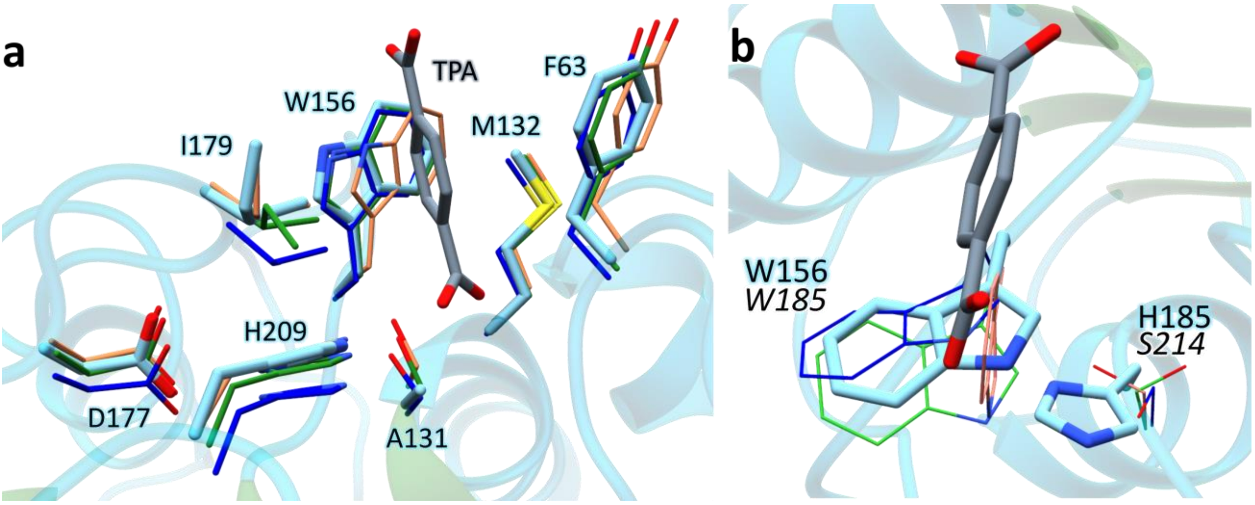

Supplementary Figure 3: Comparison of the subsite I architecture between PHL7, LCC and *Is*PETase. **a** Superimposition of subsite I of PHL7×TPA (light blue, TPA in grey) with apo structures of the homologous polyester hydrolases LCC (blue, PDB ID: 4EB0), *Is*PETase (salmon, PDB ID: 5XJH) and *Tf*Cut2 (green, 4CG1). The conformations of structurally equivalent residues are well conserved among these enzymes. *Is*PETase as a mesophilic polyester hydrolase deviates most significantly from the other enzymes that all belong to the thermophilic group. The residues of PHL7 are labeled accordingly. **b** Superimposition of PHL7×TPA (light blue, TPA in dark grey) and *Is*PETase (PDB ID: 5XG0, thin sticks). W185 as part of the subsite I π-stacking clamp of *Is*PETase adopts three distinct conformations, one in each individual molecule of the asymmetric unit (chain A: salmon; B: light green; C: blue), accompanied by different conformations of the neighboring S214. This conformational flexibility is restricted for the structurally equivalent W156 of PHL7 due to the neighboring H185. PHL7 W156 adopts a conformation most similar to conformation C of *Is*PETase. Exchange of H185S in PHL7 decreases both thermal stability and PET degradation activity.

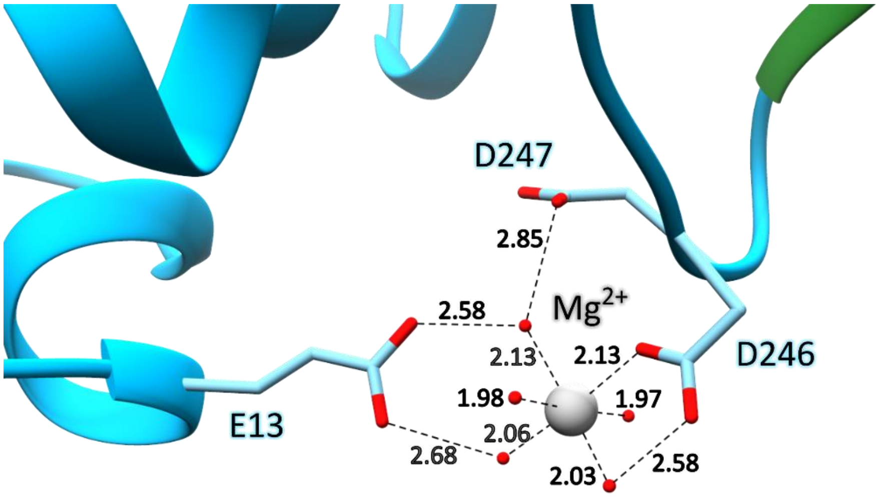

Supplementary Figure 5: Magnesium binding site of PHL7×Mg^{2+}. The carboxylic sidechain of D246 and five water molecules complex a Mg^{2+} ion in the first interaction sphere. Three of the five water molecules in

turn interact with the carboxylic sidechains of E13, D246 and D247, respectively. The binding site is ~28 Å away from the active site. All distances in Å.

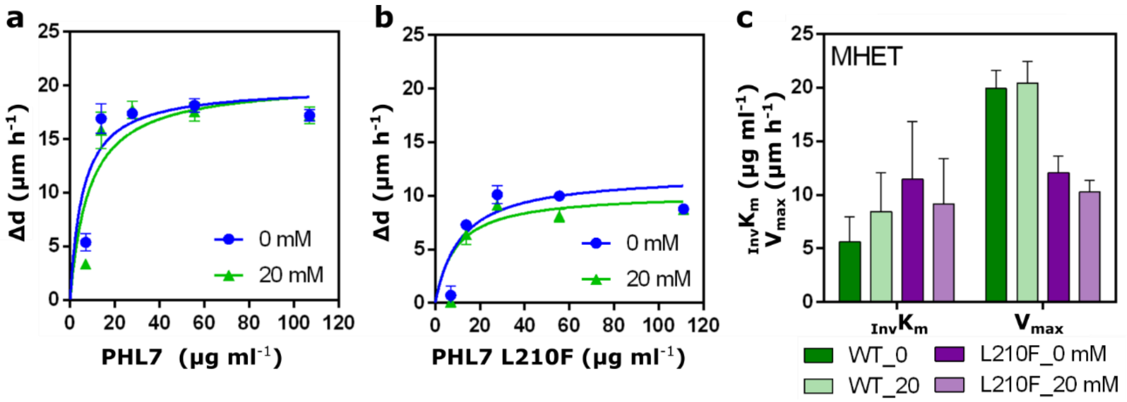

Supplementary Figure 6: Inhibition effect of MHET on PHL7 WT **a** and variant L210F **b**. The reaction rate is specified as reduction of the apparent film thickness per hour, which was measured by impedance spectroscopy[15]. An inverse Michaelis-Menten kinetic experiment was performed ranging from enzyme concentrations of 6.8 to 111 µg ml^{-1} and an inhibitor concentration of 20 mM MHET was tested. **c:** Calculated $_{Inv}K_m$ and V_{max} values for the WT and variant L210F with 0 and 20 mM MHET. $_{Inv}K_m$ describes the enzyme concentration at half-maximum reaction velocity. Mean values for n=3 replicates ± SD are shown.

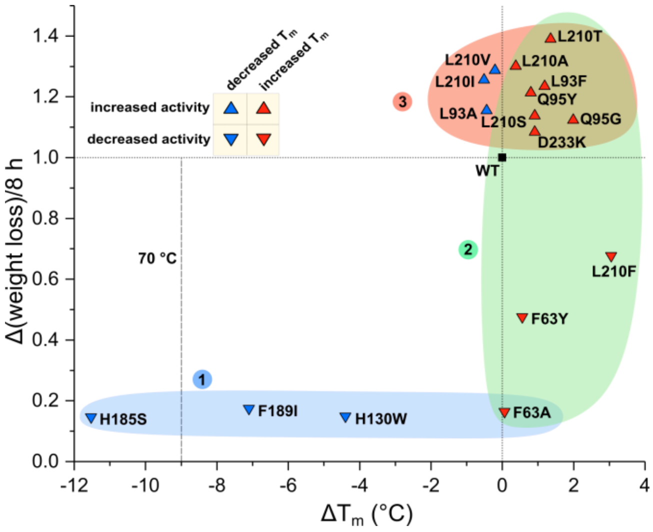

Supplementary Figure 7: Single mutants sorted by their melting point and their hydrolytic PET weight loss activity relative to PHL7 WT (black square). The triangles are color-coded (T_m decrease in blue, increase in red) and point upwards (increased weight loss activity) or downwards (decreased activity). The enzyme variants comprise three distinct classes: 1) drastically decreased activity and retained or decreased thermal stability (blue area), 2) increased thermal stability with varying activity (green) and 3) increased activity while maintaining or increasing thermal stability (red). Especially the red-green overlapping region contains double-gain variants interesting for further protein engineering. The optimal reaction temperature of 70 °C (which also resembles T_g of PET) is depicted with a dashed line.

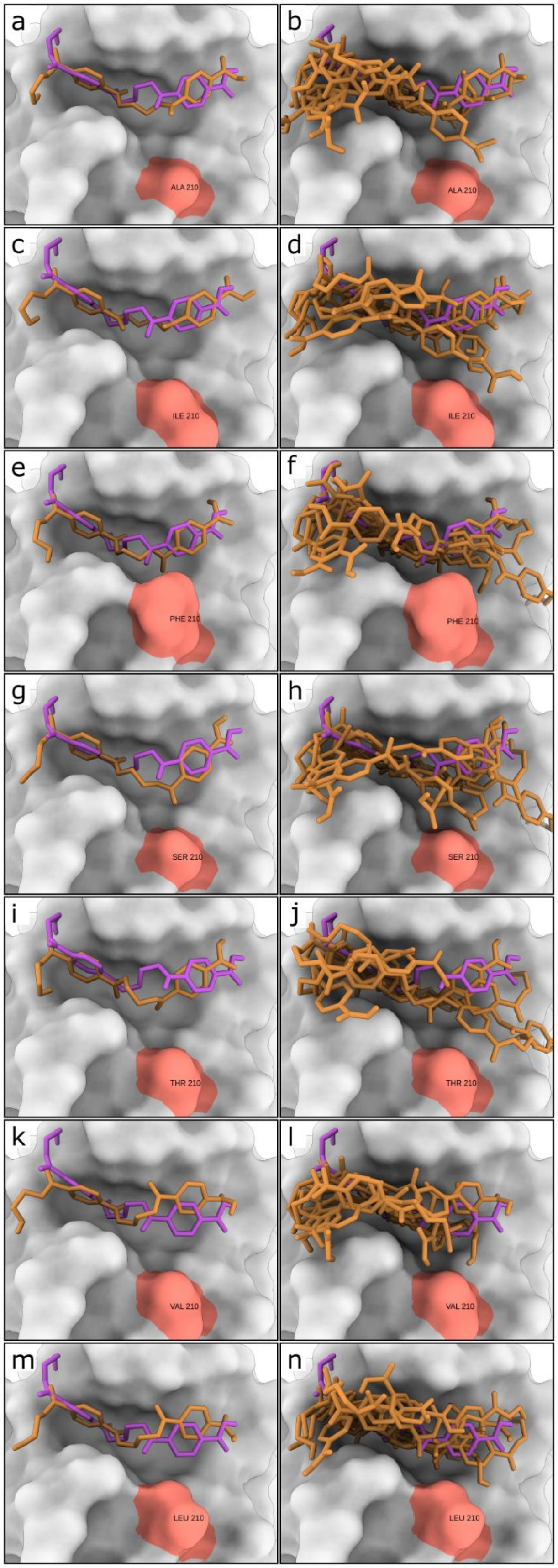

Supplementary Figure 8: Results of AutodockVina control docking calculations. Panel **a**, **c**, **e**, **g**, **i**, and k correspond to L210A, L210I, L210F, L210S, L210T, L210V, and WT, respectively. The EMT ligand lowest energy structure of Rosetta's most populated cluster is depicted in purple sticks. Shown in orange is Autodock's structurally closest prediction to Rosetta. As a comparison on the right column, panel **b**, **d**, **f**, **h**, **j**, **l**, and **n** correspond to an overlay of the nine predicted poses created with Autodock Vina.

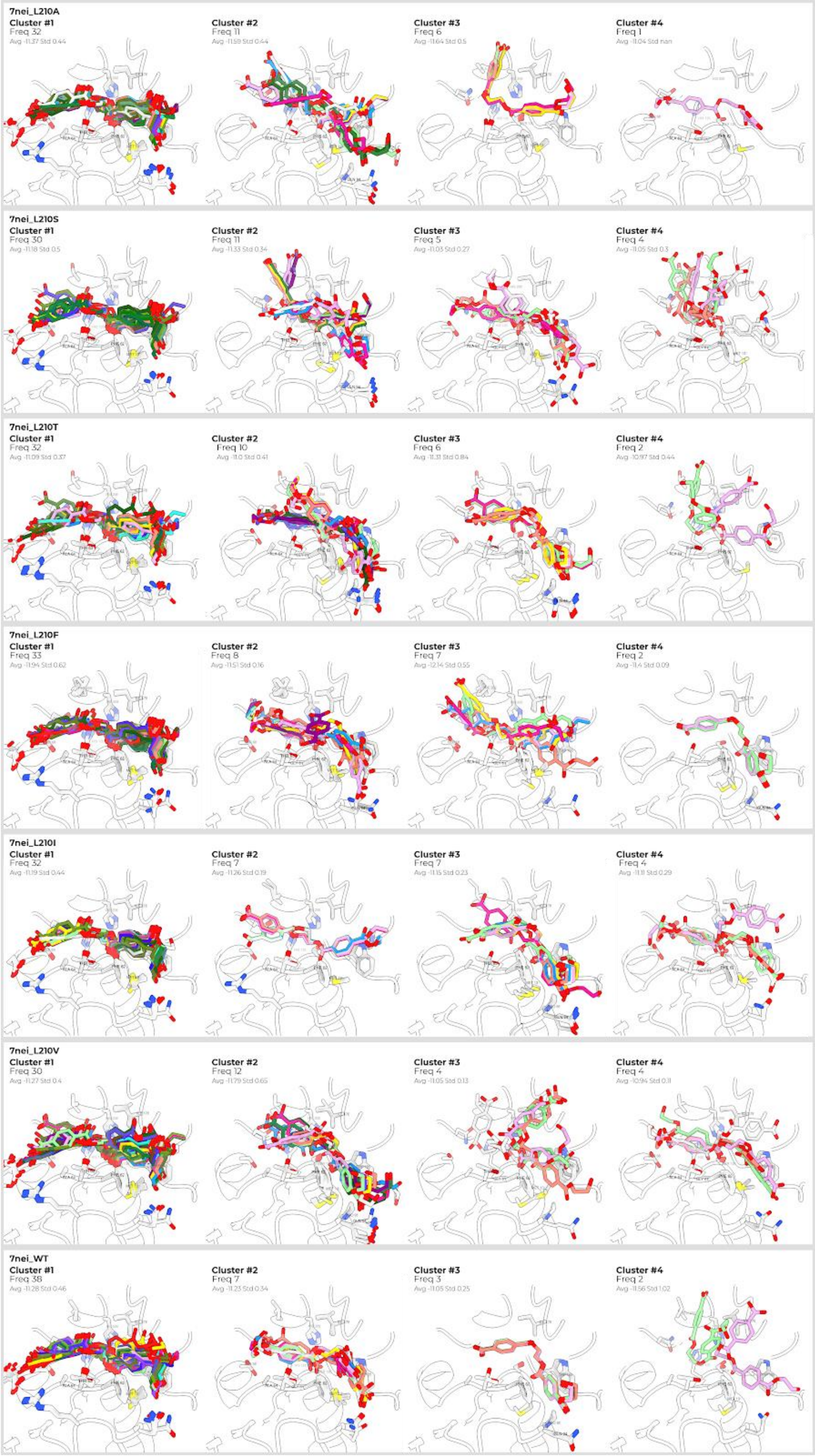
7nei_L210A
Cluster #1
Freq 32
Avg -11.37 Std 0.44
Cluster #2
Freq 11
Avg -11.59 Std 0.44
Cluster #3
Freq 6
Avg -11.64 Std 0.5
Cluster #4
Freq 1
Avg -11.04 Std nan
7nei_L210S
Cluster #1
Freq 30
Avg -11.18 Std 0.5
Cluster #2
Freq 11
Avg -11.33 Std 0.34
Cluster #3
Freq 5
Avg -11.03 Std 0.27
Cluster #4
Freq 4
Avg -11.05 Std 0.3
7nei_L210T
Cluster #1
Freq 32
Avg -11.09 Std 0.37
Cluster #2
Freq 10
Avg -11.0 Std 0.41
Cluster #3
Freq 6
Avg -11.31 Std 0.84
Cluster #4
Freq 2
Avg -10.97 Std 0.44
7nei_L210F
Cluster #1
Freq 33
Avg -11.94 Std 0.62
Cluster #2
Freq 8
Avg -11.51 Std 0.16
Cluster #3
Freq 7
Avg -12.14 Std 0.55
Cluster #4
Freq 2
Avg -11.4 Std 0.09
7nei_L210I
Cluster #1
Freq 32
Avg -11.19 Std 0.44
Cluster #2
Freq 7
Avg -11.26 Std 0.19
Cluster #3
Freq 7
Avg -11.15 Std 0.23
Cluster #4
Freq 4
Avg -11.11 Std 0.29
7nei_L210V
Cluster #1
Freq 30
Avg -11.27 Std 0.4
Cluster #2
Freq 12
Avg -11.79 Std 0.65
Cluster #3
Freq 4
Avg -11.05 Std 0.13
Cluster #4
Freq 4
Avg -10.94 Std 0.11
7nei_WT
Cluster #1
Freq 38
Avg -11.28 Std 0.46
Cluster #2
Freq 7
Avg -11.23 Std 0.34
Cluster #3
Freq 3
Avg -11.05 Std 0.25
Cluster #4
Freq 2
Avg -11.56 Std 1.02

Supplementary Figure 9: Overview of the largest molecule clusters of the 50 best energy docking models of EMT for each L210X variant tested. The model with the most likely lowest energy binding mode was selected from the largest model cluster, which is displayed on the left-hand column and contained 60-76% of the top 50 docking poses (from 40'000 generated poses in total). The Rosetta interface_delta_X score was used to estimate the energy of interaction. At the same time, each most populated cluster was aligned with the TPA crystallographic structure (8BRB) to confirm that its terephthalic ring resembled the same orientation and position. Furthermore, it is important to note that our method was able to identify alternative binding modes that have lower abundance but slightly better interface_delta_X scores in some cases (e.g. cluster #3 WT).

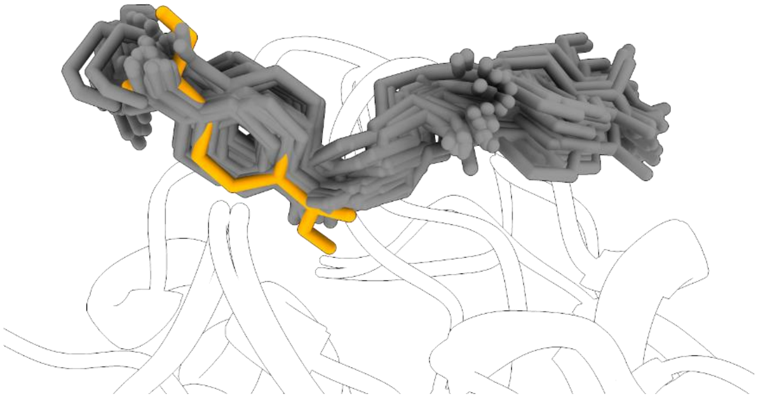

Supplementary Figure 10. Docking poses of EMT in PHL7 overlaid with the crystallized TPA molecule in PHL7. Aromatic rings of EMT molecules (gray sticks) from cluster #1 superimpose clearly with the aromatic ring of TPA (yellow sticks) bount to the crystal structure of PHL7×TPA (8BRB).

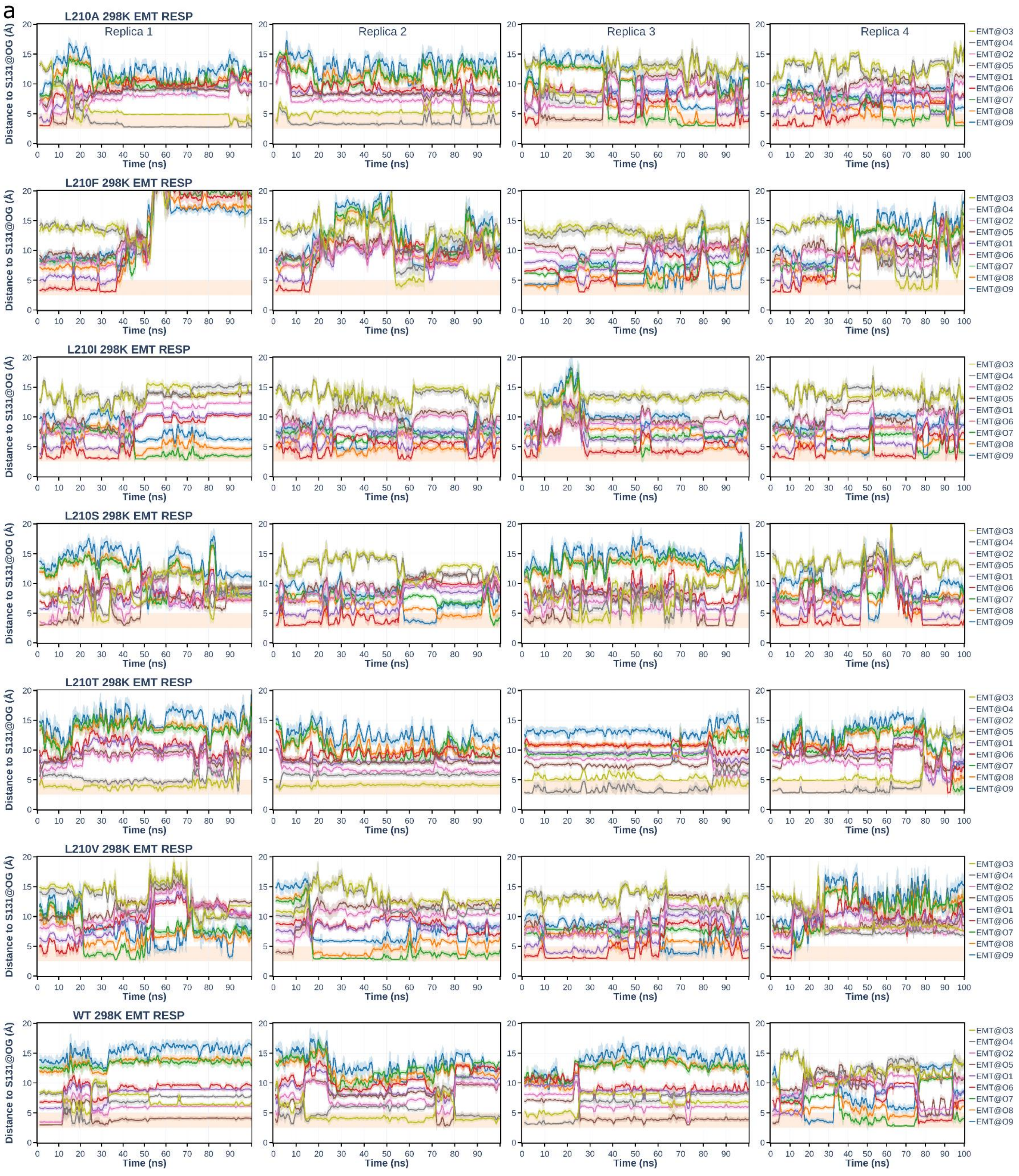
a
L210A 298K EMT RESP
L210F 298K EMT RESP
L210I 298K EMT RESP
L210S 298K EMT RESP
L210T 298K EMT RESP
L210V 298K EMT RESP
WT 298K EMT RESP
Replica 1
Replica 2
Replica 3
Replica 4
Distance to S131@OG (Å)
Time (ns)
EMT@O3
EMT@O4
EMT@O2
EMT@O5
EMT@O1
EMT@O6
EMT@O7
EMT@O8
EMT@O9

b

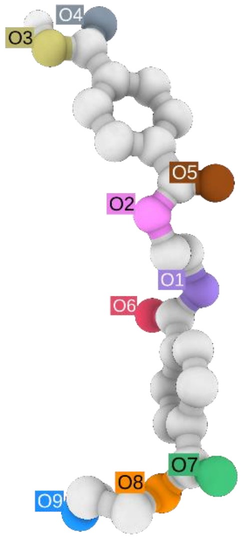

Supplementary Figure 11. MD simulation results for PHL7-EMT docking model. **a** Distance between the oxygens of the EMT molecule and the γ-oxygen of the catalytic serine (S131) over the simulation time (100 ns) for each L210X variant and the WT. For every 100 frames of the simulation the mean distance and SD were calculated. The distance considered as catalytically competent (2.5 - 5 Å) is marked as a light orange band. The S131 γ-oxygen is hydrogen-bonded with at least on oxygen of EMT over the entire time course in every MD simulation, except in two replicates for L210F and one replicate for L210V. RESP: restrained electrostatic potential. **b** Representation of the EMT ligand, with the oxygen atoms marked in the same colors as in (A) and labeled with their respective names. The oxygen atoms involved in forming the ester bonds are O1, O2 and O8.

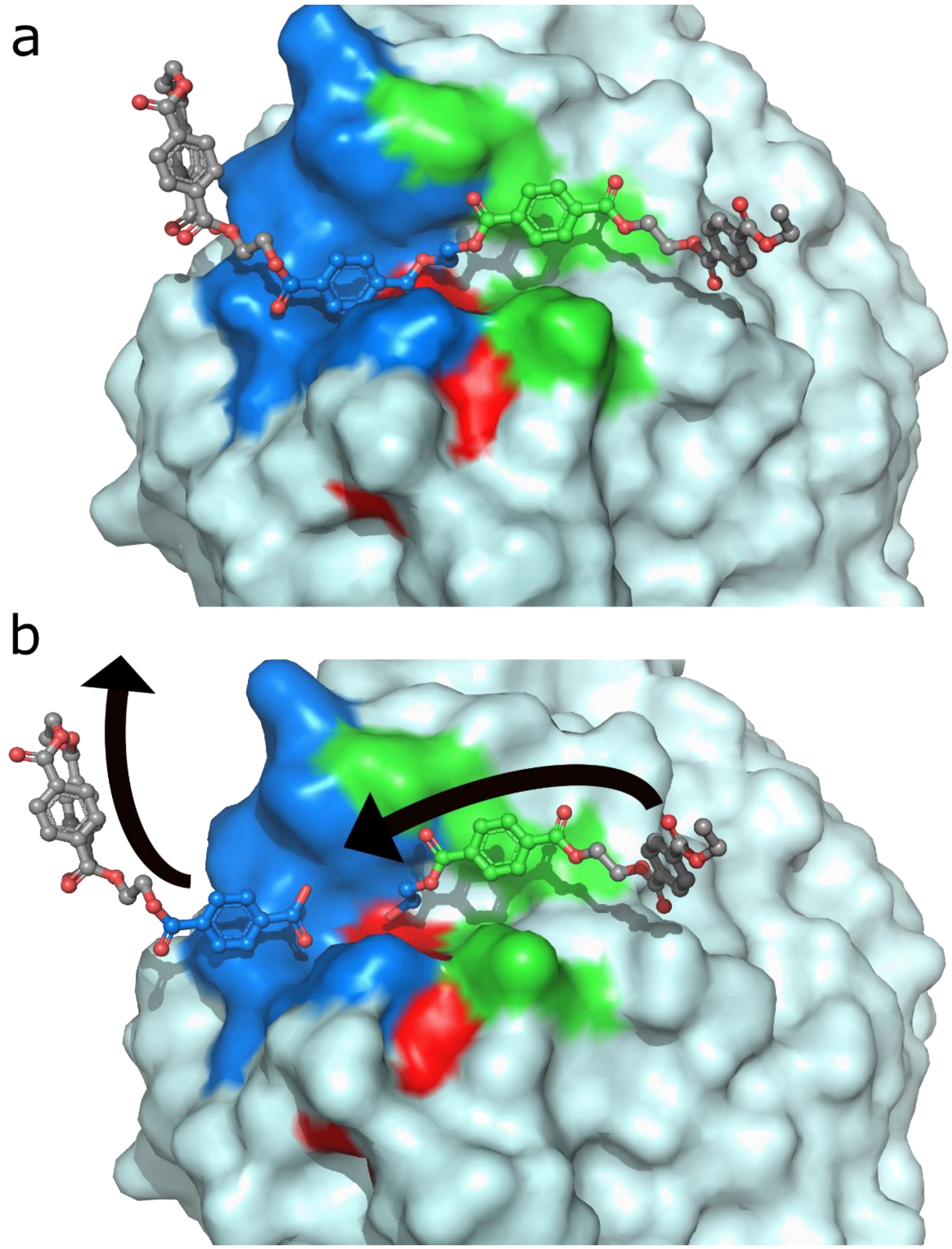

Supplementary Figure 12. Predicted binding mode of PHL7 with 2-HE(MHET)$_4$ generated using the DiffDock deep learning model. PHL7 is represented as surface model and the catalytic triad (red), subsite I (blue) and subsite II (green) are colored. The 2-HE(MHET)$_4$ is represented as sticks and sphere model. Residues of the ligand that are expected to interact with the enzyme are colored according to the respective subsite. **a** A binding mode is proposed for the 2-hydroxyethyl-(monohydroxyethyl terephthalate)$_4$, (2-HE(MHET)$_4$) ligand. **b** The proposed product after catalytic cleavage and a depiction of the hypothesized relative movement of the PET chains (indicated by black arrows) are shown.

References

1. Tian, C. *et al.* ff19SB: Amino-Acid-Specific Protein Backbone Parameters Trained against Quantum Mechanics Energy Surfaces in Solution. *Journal of chemical theory and computation* **16,** 528–552 (2020).
2. Wang, J., Wolf, R. M., Caldwell, J. W., Kollman, P. A. & Case, D. A. Development and testing of a general amber force field. *J Comput Chem* **25,** 1157–1174 (2004).
3. Anandakrishnan, R., Aguilar, B. & Onufriev, A. V. H++ 3.0: automating pK prediction and the preparation of biomolecular structures for atomistic molecular modeling and simulations. *Nucleic Acids Res* **40,** W537-41 (2012).
4. Ryckaert, J.-P., Ciccotti, G. & Berendsen, H. J. Numerical integration of the cartesian equations of motion of a system with constraints: molecular dynamics of n-alkanes. *Journal of Computational Physics* **23,** 327–341 (1977).
5. Darden, T., York, D. & Pedersen, L. Particle mesh Ewald: An N ·log(N) method for Ewald sums in large systems. *J Chem Phys* **98,** 10089–10092 (1993).
6. Wang, J., Wang, W., Kollman, P. A. & Case, D. A. Automatic atom type and bond type perception in molecular mechanical calculations. *Journal of molecular graphics & modelling* **25,** 247–260 (2006).
7. David A Case *et al.* Amber 2022, 2022.
8. Michaud-Agrawal, N., Denning, E. J., Woolf, T. B. & Beckstein, O. MDAnalysis: a toolkit for the analysis of molecular dynamics simulations. *J Comput Chem* **32,** 2319–2327 (2011).
9. Engelberger, F., Galaz-Davison, P., Bravo, G., Rivera, M. & Ramírez-Sarmiento, C. A. Developing and Implementing Cloud-Based Tutorials That Combine Bioinformatics Software, Interactive Coding, and Visualization Exercises for Distance Learning on Structural Bioinformatics. *J. Chem. Educ.* **98,** 1801–1807 (2021).
10. Trott, O. & Olson, A. J. AutoDock Vina: improving the speed and accuracy of docking with a new scoring function, efficient optimization, and multithreading. *J Comput Chem* **31,** 455–461 (2010).
11. Eberhardt, J., Santos-Martins, D., Tillack, A. F. & Forli, S. AutoDock Vina 1.2.0: New Docking Methods, Expanded Force Field, and Python Bindings. *J Chem Inf Model* **61,** 3891–3898 (2021).
12. Corso, G., Stärk, H., Jing, B., Barzilay, R. & Jaakkola, T. DiffDock: Diffusion Steps, Twists, and Turns for Molecular Docking, preprint at https://arxiv.org/pdf/2210.01776 (2022).
13. Lin, Z. *et al.* Evolutionary-scale prediction of atomic level protein structure with a language model, preprint at https://doi.org/10.1101/2022.07.20.500902 (2022).
14. Sonnendecker, C. *et al.* Low Carbon Footprint Recycling of Post-Consumer PET Plastic with a Metagenomic Polyester Hydrolase. *ChemSusChem* (2021).
15. Frank, R., Krinke, D., Sonnendecker, C., Zimmermann, W. & Jahnke, H.-G. Real-Time Noninvasive Analysis of Biocatalytic PET Degradation. *ACS Catal* **12,** 25–35 (2022).

16. Joo, S. *et al.* Structural insight into molecular mechanism of poly(ethylene terephthalate) degradation. *Nat Commun* **9,** 382 (2018).

17. Tournier, V. *et al.* An engineered PET depolymerase to break down and recycle plastic bottles. *Nature* **580,** 216–219 (2020).

18. Chen, C.-C. *et al.* General features to enhance enzymatic activity of poly(ethylene terephthalate) hydrolysis. *Nat Catal* **4,** 425–430 (2021).

19. Roth, C. *et al.* Structural and functional studies on a thermostable polyethylene terephthalate degrading hydrolase from Thermobifida fusca. *Appl Microbiol Biotechnol* **98,** 7815–7823 (2014).

20. Guo, B. *et al.* Conformational Selection in Biocatalytic Plastic Degradation by PETase. *ACS Catal* **12,** 3397–3409 (2022).
