# Supplementary material for: Structure and function of the metagenomic plastic-degrading polyester hydrolase PHL7 bound to its product
Source: Nat Commun. 2023 Apr 5;14:1905. doi: 10.1038/s41467-023-37415-x (PMC10076380; doi:10.1038/s41467-023-37415-x)
Supplement: Supplementary file 2 — Reporting Summary [file 41467_2023_37415_MOESM2_ESM.pdf]

nature portfolio

Corresponding author(s): Christian Sonnendecker, Wolfgang Zimmermann, Norbert Sträter

Last updated by author(s): Feb 23, 2023

# Reporting Summary

Nature Portfolio wishes to improve the reproducibility of the work that we publish. This form provides structure for consistency and transparency in reporting. For further information on Nature Portfolio policies, see our Editorial Policies and the Editorial Policy Checklist.

## Statistics

For all statistical analyses, confirm that the following items are present in the figure legend, table legend, main text, or Methods section.

| n/a | Confirmed | |
|---|---|---|
| ☐ | ☒ | The exact sample size (*n*) for each experimental group/condition, given as a discrete number and unit of measurement |
| ☐ | ☒ | A statement on whether measurements were taken from distinct samples or whether the same sample was measured repeatedly |
| ☐ | ☒ | The statistical test(s) used AND whether they are one- or two-sided<br>*Only common tests should be described solely by name; describe more complex techniques in the Methods section.* |
| ☒ | ☐ | A description of all covariates tested |
| ☒ | ☐ | A description of any assumptions or corrections, such as tests of normality and adjustment for multiple comparisons |
| ☐ | ☒ | A full description of the statistical parameters including central tendency (e.g. means) or other basic estimates (e.g. regression coefficient) AND variation (e.g. standard deviation) or associated estimates of uncertainty (e.g. confidence intervals) |
| ☐ | ☒ | For null hypothesis testing, the test statistic (e.g. *F*, *t*, *r*) with confidence intervals, effect sizes, degrees of freedom and *P* value noted<br>*Give P values as exact values whenever suitable.* |
| ☒ | ☐ | For Bayesian analysis, information on the choice of priors and Markov chain Monte Carlo settings |
| ☒ | ☐ | For hierarchical and complex designs, identification of the appropriate level for tests and full reporting of outcomes |
| ☒ | ☐ | Estimates of effect sizes (e.g. Cohen's *d*, Pearson's *r*), indicating how they were calculated |

*Our web collection on statistics for biologists contains articles on many of the points above.*

## Software and code

Policy information about availability of computer code

| | |
|---|---|
| Data collection | Prometheus NT.48 2.3.1 control software (for determination of melting points)<br>Gen5 data analysis software for BioTek Synergy Mx Microplate Reader<br>IMATadvanced v2021 (self-developed for mulitplexing EIS data)<br>CrysAlis (Pro) software 41.108a (for indexing, integrating and reducing datasets from crystallographic experiments at home source)<br>Rosetta3 (molecular docking, custom Rosetta3 XML script) |

| | |
|---|---|
| Data analysis | Microsoft Excel 2019 (weight loss analysis)<br>MATLAB script V7.R14 with the Nelder−Mead method (analysis of EIS data)<br>AIMLESS 0.7.7 (scaling crystallographic data)<br>PHENIX 1.20_4459 (refinement of crystallographic data)<br>COOT 0.9.7 (model building for crystallographic data)<br>GRADE 1.2.19 webserver (calculation of TPA restraints)<br>UCSF Chimera 1.13.1_b41965 (molecular analyses and graphics)<br>R stats 4.1 (dist() function and Ward.D2 (cluster distance matrix)<br>factoextra V1.0.7 and ggplot2 V3.3.5 (visualization of clustering results (fviz cluster function))<br>Rosetta 3.12 ddG mover (per residue energetic contribution to ligang binding)<br>Gaussian 16 (quantum mechnanical calculations)<br>Graphpad Prism 7 (statistical analysis) |

For manuscripts utilizing custom algorithms or software that are central to the research but not yet described in published literature, software must be made available to editors and reviewers. We strongly encourage code deposition in a community repository (e.g. GitHub). See the Nature Portfolio guidelines for submitting code & software for further information.

## Data

Policy information about availability of data

All manuscripts must include a data availability statement. This statement should provide the following information, where applicable:

- Accession codes, unique identifiers, or web links for publicly available datasets
- A description of any restrictions on data availability
- For clinical datasets or third party data, please ensure that the statement adheres to our policy

The data generated in this study are available within the article and the supplementary information. Crystallographic structures have been deposited at the Protein Data Bank (https://www.rcsb.org/) under accession numbers: 8BRA and 8BRB. Further data from our Rosetta simulations and DFT calculations are available upon reasonable request.

## Human research participants

Policy information about studies involving human research participants and Sex and Gender in Research.

| | |
|---|---|
| Reporting on sex and gender | N/A |
| Population characteristics | N/A |
| Recruitment | N/A |
| Ethics oversight | N/A |

Note that full information on the approval of the study protocol must also be provided in the manuscript.

# Field-specific reporting

Please select the one below that is the best fit for your research. If you are not sure, read the appropriate sections before making your selection.

[x] Life sciences [ ] Behavioural & social sciences [ ] Ecological, evolutionary & environmental sciences

For a reference copy of the document with all sections, see nature.com/documents/nr-reporting-summary-flat.pdf

# Life sciences study design

All studies must disclose on these points even when the disclosure is negative.

| | |
|---|---|
| Sample size | For enzymatical assays and Tm assessment, three replicates were performed (n=5 for impedance data) to determine mean and standard deviation. We choose a sample size of n=3, based on the practical constraints of the experiment. it is a common sample size used in similar experiments in the literature. |
| Data exclusions | no data has been excluded |
| Replication | All in vitro experiments with explicit standard deviation (SD) were performed in triplicates. Attempts at independent experiments were successful. WT PHL7 (six times) and L210T activity data has been confirmed in independent experiments. |
| Randomization | not relevant |
| Blinding | For impedance-spectroscopical analysis, the analytical team who analyzed the degradation rates were blind to the samples |

# Reporting for specific materials, systems and methods

We require information from authors about some types of materials, experimental systems and methods used in many studies. Here, indicate whether each material, system or method listed is relevant to your study. If you are not sure if a list item applies to your research, read the appropriate section before selecting a response.

## Materials & experimental systems

| n/a | Involved in the study |
|---|---|
| [x] | [ ] Antibodies |
| [x] | [ ] Eukaryotic cell lines |
| [x] | [ ] Palaeontology and archaeology |
| [x] | [ ] Animals and other organisms |
| [x] | [ ] Clinical data |
| [x] | [ ] Dual use research of concern |

## Methods

| n/a | Involved in the study |
|---|---|
| [x] | [ ] ChIP-seq |
| [x] | [ ] Flow cytometry |
| [x] | [ ] MRI-based neuroimaging |
